# Lithium manganese(II) diaqua­boro­phosphate monohydrate

**DOI:** 10.1107/S1600536808018898

**Published:** 2008-07-05

**Authors:** Rong-Chuan Zhuang, Xue-Yun Chen, Jin-Xiao Mi

**Affiliations:** a Department of Materials Science and Engineering, College of Materials, Xiamen University, Xiamen 361005, Fujian Province, People’s Republic of China; b Department of Chemistry and Biochemical Engineering, College of Chemistry and Chemical Engineering, Xiamen University, Xiamen 361005, Fujian Province, People’s Republic of China

## Abstract

The title compound, LiMn(H_2_O)_2_[BP_2_O_8_]·H_2_O, is built up of an open framework of helical borophosphate ribbons inter­connected by MnO_4_(H_2_O)_2_ octa­hedra, forming one-dimensional channels along [001] occupied by Li^+^ cations and disordered H_2_O mol­ecules (site occupancy 0.5). The Li cations reside in two partially occupied sites [occupancies = 0.42 (3) and 0.289 (13)] near the helices.

## Related literature

For related structures, see: Boy & Kniep (2001*a* ▶,*b* ▶) for LiCu(H_2_O)_2_[BP_2_O_8_]·(H_2_O) and LiZn(H_2_O)_2_[BP_2_O_8_]·H_2_O; Ge *et al.* (2003 ▶) for LiCd(H_2_O)_2_[BP_2_O_8_]·H_2_O; Lin *et al.* (2008 ▶) for LiMg(H_2_O)_2_[BP_2_O_8_]·H_2_O. For related literature, see: Ewald *et al.* (2006 ▶); Kniep *et al.* (1997 ▶).

## Experimental

### 

#### Crystal data

LiMn(H_2_O)_2_[BP_2_O_8_]·H_2_O
*M* *_r_* = 316.68
Hexagonal, 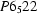

*a* = 9.5765 (4) Å
*c* = 15.857 (1) Å
*V* = 1259.4 (1) Å^3^
*Z* = 6
Mo *K*α radiation
μ = 2.01 mm^−1^
*T* = 295 (2) K
0.16 × 0.12 × 0.12 mm

#### Data collection

Rigaku AFC-7 CCD diffractometer
Absorption correction: multi-scan (*CrystalClear*; Rigaku, 2005 ▶) *T*_min_ = 0.740, *T*_max_ = 0.795
9731 measured reflections
1230 independent reflections
1223 reflections with *I* > 2σ(*I*)
*R*_int_ = 0.032

#### Refinement

*R*[*F* ^2^ > 2σ(*F* ^2^)] = 0.040
*wR*(*F* ^2^) = 0.097
*S* = 1.19
1230 reflections
85 parameters
1 restraint
Only H-atom coordinates refined
Δρ_max_ = 0.61 e Å^−3^
Δρ_min_ = −0.44 e Å^−3^
Absolute structure: Flack (1983 ▶), 443 Friedel pairs
Flack parameter: −0.01 (4)

### 

Data collection: *CrystalClear* (Rigaku, 2005 ▶); cell refinement: *CrystalClear*; data reduction: *CrystalClear*; program(s) used to solve structure: *SHELXS97* (Sheldrick, 2008 ▶); program(s) used to refine structure: *SHELXL97* (Sheldrick, 2008 ▶); molecular graphics: *DIAMOND* (Brandenburg, 2005 ▶) and *ATOMS* (Dowty, 2004 ▶); software used to prepare material for publication: *SHELXL97*.

## Supplementary Material

Crystal structure: contains datablocks I, global. DOI: 10.1107/S1600536808018898/mg2052sup1.cif

Structure factors: contains datablocks I. DOI: 10.1107/S1600536808018898/mg2052Isup2.hkl

Additional supplementary materials:  crystallographic information; 3D view; checkCIF report

## Figures and Tables

**Table:** 

Mn1—O4^i^	2.133 (3)
Mn1—O3	2.139 (3)
Mn1—O5	2.311 (4)
P1—O3	1.504 (3)
P1—O4	1.510 (3)
P1—O1^ii^	1.553 (3)
P1—O2^iii^	1.560 (2)
O5—H1	0.82 (7)
O5—H2	0.81 (2)
B1—O1^iv^	1.463 (4)
B1—O2^ii^	1.470 (4)
Li1—O5^ii^	2.111 (4)
Li1—O3^ii^	2.112 (17)
Li2—O6^v^	1.95 (3)
Li2—O6^vi^	1.98 (3)
Li2—O4^v^	2.11 (3)
Li2—O5^vii^	2.18 (3)

**Table:** 

B1^viii^—O1—P1^ii^	129.4 (2)
B1—O2—P1^ix^	131.1 (2)
P1—O3—Mn1	128.38 (17)

Symmetry codes: (i) 
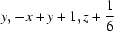
; (ii) 
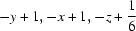
; (iii) 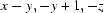; (iv) 
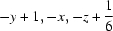
; (v) 
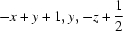
; (vi) 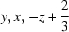; (vii) 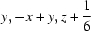; (viii) 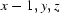; (ix) 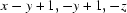.

**Table 2:** Hydrogen-bond geometry (Å, °)

*D*—H⋯*A*	*D*—H	H⋯*A*	*D*⋯*A*	*D*—H⋯*A*
O5—H1⋯O4^x^	0.83 (7)	2.09 (7)	2.878 (5)	159.80
O5—H2⋯O2^i^	0.81 (4)	2.09 (5)	2.845 (5)	155.74

Symmetry codes: (i) 
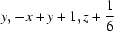
; (x) 
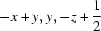
.

**Fig. 1.**
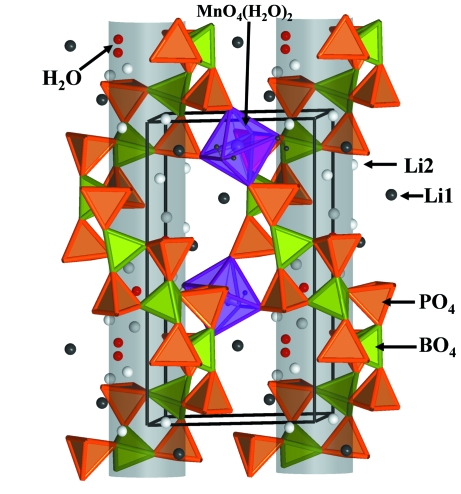
Linkage of borophosphate helices in LiMn(H2O)2[BP2O8].H2O through MnO4(H2O)2 octahedra (BO4, green tetrahedra; PO4, orange tetrahedra; MnO6, violet octahedra; Li, black spheres; H2O, red spheres).

**Fig. 2.**
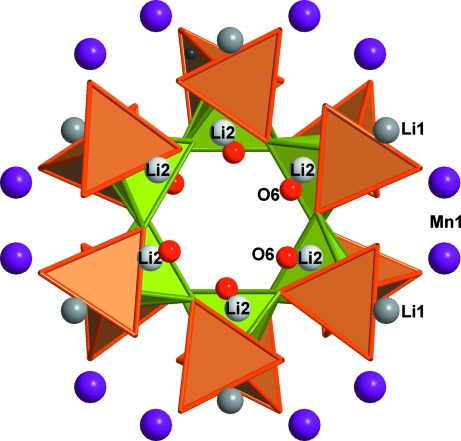
Section of LiMn(H2O)2[BP2O8].H2O viewed along the c axis (colour scheme as in Fig. 1).

**Fig. 3.**
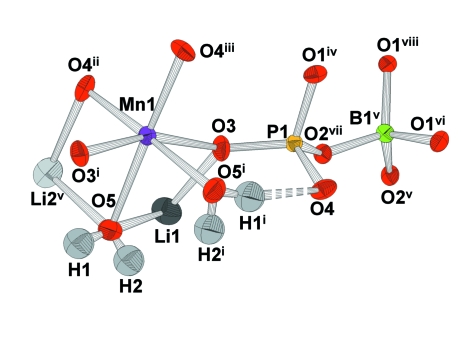
Coordination environment of Mn, B, and P atoms, with displacement ellipsoids drawn at the 50% probability level (symmetry codes: (i) -x+y, y, 1/2-z; (ii) 1-x, 1-x+y, 1/3-z; (iii) y,1-x+y, 1/6+z; (iv) 1-y,1-x, 1/6-z; (v) x-y, x,-1/6+z; (vi) 1+x-y, 1-y,-z, (vii) x-y, 1-y,-z; (viii) 1+x-y,1+x,-1/6+z).

## supplementary crystallographic information

### Comment

A large family of compounds contains helical borophosphate anions _∞_^1^[BP_2_O_8_]^3-^ with various combinations of metal cations (*M*^I^*M*^II^, *M*_0.5_^I^*M*^II^, *M*^III^) (Kniep *et al.*, 1997; Ewald *et al.*, 2006). To date, the only Li-containing members are Li*M*^II^(H_2_O)_2_[BP_2_O_8_]^.^H_2_O (*M*^II^ = Cu, Zn, Cd, Mg) (Boy & Kniep, 2001a, 2001b; Ge *et al.*, 2003; Lin *et al.*, 2008). The structure of LiMn(H_2_O)_2_[BP_2_O_8_]^.^H_2_O is reported here.

The borophosphate helices, built up of four-membered rings of alternating BO_4_ and PO_4_ tetrahedra, extend along the 6_5_ screw axis (Fig. 1 and 2). These helices are interconnected by Jahn-Teller-distorted Mn^2+^-centred octahedra, with four oxygen atoms (O3, O4) from PO_4_ groups and two (O5) from water molecules at the vertices (Fig. 3). Unlike the compounds containing Cu and Zn (Boy & Kniep, 2001a, 2001b) but similar to those containing Cd and Mg (Ge *et al.*, 2003; Lin *et al.*, 2008), there are two distinct Li sites: Li1 is close to the outer wall of the borophosphate helices and Li2 is situated at the free loops (inner wall) of the helices. The sum of occupancies of these Li sites refines to almost unity, as required to maintain charge neutrality in the compound.

### Experimental

LiMn(H_2_O)_2_[BP_2_O_8_].H_2_O was obtained in the presence of boric acid as a flux. A mixture of 0.1149 g MnCO_3_, 1.484 g H_3_BO_3_, and 0.6235 g LiH_2_PO_4_ was ground to a homogeneous powder, which was transferred to a teflon autoclave with 10 ml inline (degree of filling 10%) where it was heated at 443 K for four days.

### Refinement

The hydrogen atoms connected to O5 were located from difference Fourier maps with displacement parameters fixed as 1.2*U(O5), whereas those connected to O6 belonging to the disordered water molecules were not located. The sum of the occupancies of Li sites was restrained to maintain charge neutrality within the entire compound. The occupancy of the O6 site associated with the disordered water molecules was fixed at 0.5.

### Crystal data

**Table:**

LiMn(H_2_O)_2_[BP_2_O_8_]·H_2_O	*D*_x_ = 2.505 Mg m^−^^3^
*M**_r_* = 316.68	Mo *K*α radiation, λ = 0.71073 Å
Hexagonal, *P*6_5_22	Cell parameters from 6263 reflections
Hall symbol: P 65 2 (0 0 1)	θ = 2.5–33.2°
*a* = 9.5765 (4) Å	µ = 2.01 mm^−^^1^
*c* = 15.857 (1) Å	*T* = 295 K
*V* = 1259.4 (1) Å^3^	Hexagonal bipyramid, pale pink
*Z* = 6	0.16 × 0.12 × 0.12 mm
*F*(000) = 942	

### Data collection

**Table:**

Rigaku AFC-7 CCD diffractometer	1230 independent reflections
Radiation source: fine-focus sealed tube	1223 reflections with *I* > 2σ(*I*)
graphite	*R*_int_ = 0.032
Detector resolution: 14.6306 pixels mm^-1^	θ_max_ = 30.0°, θ_min_ = 2.5°
thin–slice Δφ=0.6 & Δω=0.6 scans	*h* = −13→13
Absorption correction: multi-scan (*CrystalClear*; Rigaku, 2005)	*k* = −13→12
*T*_min_ = 0.740, *T*_max_ = 0.795	*l* = −19→22
9731 measured reflections	

### Refinement

**Table:**

Refinement on *F*^2^	Hydrogen site location: difference Fourier map
Least-squares matrix: full	Only H-atom coordinates refined
*R*[*F*^2^ > 2σ(*F*^2^)] = 0.040	*w* = 1/[σ^2^(*F*_o_^2^) + (0.008*P*)^2^ + 5.1269*P*] where *P* = (*F*_o_^2^ + 2*F*_c_^2^)/3
*wR*(*F*^2^) = 0.097	(Δ/σ)_max_ < 0.001
*S* = 1.19	Δρ_max_ = 0.62 e Å^−^^3^
1230 reflections	Δρ_min_ = −0.44 e Å^−^^3^
85 parameters	Extinction correction: *SHELXL97* (Sheldrick, 2008), Fc^*^=kFc[1+0.001xFc^2^λ^3^/sin(2θ)]^-1/4^
1 restraint	Extinction coefficient: 0.0054 (19)
Primary atom site location: structure-invariant direct methods	Absolute structure: Flack (1983), 443 Friedel pairs
Secondary atom site location: difference Fourier map	Flack parameter: −0.01 (4)

### Special details

**Table:**

Geometry. All esds (except the esd in the dihedral angle between two l.s. planes) are estimated using the full covariance matrix. The cell esds are taken into account individually in the estimation of esds in distances, angles and torsion angles; correlations between esds in cell parameters are only used when they are defined by crystal symmetry. An approximate (isotropic) treatment of cell esds is used for estimating esds involving l.s. planes.
Refinement. Refinement of *F*^2^ against ALL reflections. The weighted *R*-factor *wR* and goodness of fit *S* are based on *F*^2^, conventional *R*-factors *R* are based on *F*, with *F* set to zero for negative *F*^2^. The threshold expression of *F*^2^ > σ(*F*^2^) is used only for calculating *R*-factors(gt) *etc*. and is not relevant to the choice of reflections for refinement. *R*-factors based on *F*^2^ are statistically about twice as large as those based on *F*, and *R*- factors based on ALL data will be even larger.

### Fractional atomic coordinates and isotropic or equivalent isotropic displacement parameters (Å^2^)

**Table:**

	*x*	*y*	*z*	*U*_iso_*/*U*_eq_	Occ. (<1)
Mn1	0.44888 (4)	0.89775 (9)	0.2500	0.0163 (2)	
P1	0.61636 (10)	0.83327 (10)	0.08453 (6)	0.0134 (2)	
O1	0.0204 (3)	0.2129 (3)	0.06593 (16)	0.0181 (5)	
O2	0.7681 (3)	0.1804 (3)	0.01267 (14)	0.0158 (5)	
O3	0.4860 (3)	0.8589 (4)	0.12112 (17)	0.0227 (6)	
O4	0.6237 (4)	0.6903 (3)	0.11938 (17)	0.0228 (6)	
O5	0.1884 (4)	0.7081 (4)	0.2127 (2)	0.0340 (8)	
O6	0.9000 (19)	0.8166 (12)	0.2717 (7)	0.079 (3)*	0.50
B1	0.8493 (3)	0.1507 (3)	0.0833	0.0140 (9)	
Li1	0.2428 (18)	0.7572 (18)	0.0833	0.034 (4)	0.42 (3)
Li2	0.899 (4)	0.763 (3)	0.3479 (16)	0.034 (4)	0.289 (13)
H1	0.133 (8)	0.683 (7)	0.256 (4)	0.041*	
H2	0.179 (7)	0.620 (4)	0.218 (4)	0.041*	

### Atomic displacement parameters (Å^2^)

**Table:**

	*U*^11^	*U*^22^	*U*^33^	*U*^12^	*U*^13^	*U*^23^
Mn1	0.0170 (3)	0.0165 (4)	0.0153 (3)	0.00826 (18)	0.0028 (2)	0.000
P1	0.0153 (4)	0.0131 (4)	0.0113 (3)	0.0068 (3)	0.0011 (3)	−0.0005 (3)
O1	0.0139 (11)	0.0196 (12)	0.0200 (12)	0.0077 (10)	−0.0012 (9)	−0.0060 (9)
O2	0.0195 (12)	0.0185 (11)	0.0106 (9)	0.0104 (10)	−0.0029 (9)	−0.0024 (8)
O3	0.0222 (14)	0.0306 (15)	0.0177 (12)	0.0151 (12)	0.0028 (10)	−0.0046 (11)
O4	0.0348 (16)	0.0150 (12)	0.0177 (11)	0.0117 (11)	−0.0008 (12)	0.0022 (10)
O5	0.0280 (17)	0.0233 (15)	0.0393 (17)	0.0041 (13)	0.0118 (14)	−0.0065 (14)
B1	0.0157 (17)	0.0157 (17)	0.011 (2)	0.0083 (19)	0.0013 (16)	0.0013 (16)
Li1	0.036 (7)	0.036 (7)	0.023 (8)	0.012 (8)	0.003 (6)	0.003 (6)
Li2	0.036 (7)	0.036 (7)	0.023 (8)	0.012 (8)	0.003 (6)	0.003 (6)

### Geometric parameters (Å, °)

**Table:**

Mn1—O4^i^	2.133 (3)	O5—H1	0.82 (7)
Mn1—O4^ii^	2.133 (3)	O5—H2	0.81 (2)
Mn1—O3	2.139 (3)	O6—O6^ix^	0.71 (2)
Mn1—O3^iii^	2.139 (3)	O6—Li2	1.31 (3)
Mn1—O5	2.311 (4)	O6—Li2^ix^	1.95 (3)
Mn1—O5^iii^	2.311 (3)	O6—Li2^xii^	1.98 (3)
P1—O3	1.504 (3)	O6—Li2^x^	2.45 (3)
P1—O4	1.510 (3)	O6—Li1^xiii^	2.53 (3)
P1—O1^iv^	1.553 (3)	B1—O1^xiv^	1.463 (4)
P1—O2^v^	1.560 (2)	B1—O1^xv^	1.463 (4)
O1—B1^vi^	1.463 (4)	B1—O2^iv^	1.470 (4)
O1—P1^iv^	1.553 (3)	Li1—O5^iv^	2.111 (4)
O1—Li2^vii^	2.65 (3)	Li1—O3^iv^	2.112 (17)
O2—B1	1.470 (4)	Li2—O6^ix^	1.95 (3)
O2—P1^viii^	1.560 (2)	Li2—O6^xii^	1.98 (3)
O3—Li1	2.112 (17)	Li2—O4^ix^	2.11 (3)
O4—Li2^ix^	2.11 (3)	Li2—O5^xiii^	2.18 (3)
O4—Mn1^x^	2.133 (3)	Li2—Li2^xii^	2.30 (6)
O5—Li1	2.111 (4)	Li2—O6^i^	2.45 (3)
O5—Li2^xi^	2.18 (3)		
			
O4^i^—Mn1—O4^ii^	97.89 (17)	O2—B1—O2^iv^	102.6 (4)
O4^i^—Mn1—O3	100.17 (11)	O5^iv^—Li1—O5	177.6 (17)
O4^ii^—Mn1—O3	91.22 (11)	O5^iv^—Li1—O3^iv^	85.4 (4)
O4^i^—Mn1—O3^iii^	91.22 (11)	O5—Li1—O3^iv^	96.0 (5)
O4^ii^—Mn1—O3^iii^	100.17 (11)	O5^iv^—Li1—O3	96.0 (5)
O3—Mn1—O3^iii^	162.68 (17)	O5—Li1—O3	85.4 (4)
O4^i^—Mn1—O5	178.14 (13)	O3^iv^—Li1—O3	112.6 (14)
O4^ii^—Mn1—O5	83.95 (13)	O5^iv^—Li1—O6^ii^	80.8 (8)
O3—Mn1—O5	80.01 (12)	O5—Li1—O6^ii^	96.8 (9)
O3^iii^—Mn1—O5	88.19 (12)	O3^iv^—Li1—O6^ii^	122.4 (7)
O4^i^—Mn1—O5^iii^	83.95 (13)	O3—Li1—O6^ii^	124.3 (8)
O4^ii^—Mn1—O5^iii^	178.14 (14)	O5^iv^—Li1—O6^xi^	96.8 (9)
O3—Mn1—O5^iii^	88.19 (12)	O5—Li1—O6^xi^	80.8 (8)
O3^iii^—Mn1—O5^iii^	80.01 (12)	O3^iv^—Li1—O6^xi^	124.3 (8)
O5—Mn1—O5^iii^	94.2 (2)	O3—Li1—O6^xi^	122.4 (7)
O3—P1—O4	115.17 (17)	O6^ii^—Li1—O6^xi^	16.0 (5)
O3—P1—O1^iv^	111.97 (16)	O6—Li2—O6^ix^	10.8 (10)
O4—P1—O1^iv^	104.62 (16)	O6—Li2—O6^xii^	90.8 (17)
O3—P1—O2^v^	105.62 (15)	O6^ix^—Li2—O6^xii^	101.7 (15)
O4—P1—O2^v^	111.81 (15)	O6—Li2—O4^ix^	119.9 (19)
O1^iv^—P1—O2^v^	107.54 (14)	O6^ix^—Li2—O4^ix^	110.3 (14)
B1^vi^—O1—P1^iv^	129.4 (2)	O6^xii^—Li2—O4^ix^	138.2 (14)
B1—O2—P1^viii^	131.1 (2)	O6—Li2—O5^xiii^	117.8 (18)
P1—O3—Mn1	128.38 (17)	O6^ix^—Li2—O5^xiii^	114.8 (14)
Li1—O5—H1	157 (4)	O6^xii^—Li2—O5^xiii^	102.8 (13)
Li2^xi^—O5—H1	93 (4)	O4^ix^—Li2—O5^xiii^	87.8 (11)
Mn1—O5—H1	107 (4)	O6—Li2—O6^i^	104.2 (18)
Li1—O5—H2	103 (4)	O6^ix^—Li2—O6^i^	115.0 (14)
Li2^xi^—O5—H2	162 (4)	O6^xii^—Li2—O6^i^	13.8 (7)
Mn1—O5—H2	107 (4)	O4^ix^—Li2—O6^i^	125.5 (12)
H1—O5—H2	84 (5)	O5^xiii^—Li2—O6^i^	99.1 (11)
Li2—O6—Li2^ix^	146 (2)	O6—Li2—O1^xvi^	100.4 (16)
O1^xiv^—B1—O1^xv^	103.7 (4)	O6^ix^—Li2—O1^xvi^	100.1 (12)
O1^xiv^—B1—O2	113.70 (14)	O6^xii^—Li2—O1^xvi^	89.1 (11)
O1^xv^—B1—O2	111.75 (14)	O4^ix^—Li2—O1^xvi^	59.9 (8)
O1^xiv^—B1—O2^iv^	111.75 (14)	O5^xiii^—Li2—O1^xvi^	139.4 (13)
O1^xv^—B1—O2^iv^	113.70 (14)	O6^i^—Li2—O1^xvi^	83.4 (9)

Symmetry codes: (i) *y*, −*x*+*y*+1, *z*+1/6; (ii) −*x*+1, −*x*+*y*+1, −*z*+1/3; (iii) −*x*+*y*, *y*, −*z*+1/2; (iv) −*y*+1, −*x*+1, −*z*+1/6; (v) *x*−*y*, −*y*+1, −*z*; (vi) *x*−1, *y*, *z*; (vii) −*y*+1, *x*−*y*, *z*−1/3; (viii) *x*−*y*+1, −*y*+1, −*z*; (ix) −*x*+*y*+1, *y*, −*z*+1/2; (x) *x*−*y*+1, *x*, *z*−1/6; (xi) *x*−*y*, *x*, *z*−1/6; (xii) *y*, *x*, −*z*+2/3; (xiii) *y*, −*x*+*y*, *z*+1/6; (xiv) −*y*+1, −*x*, −*z*+1/6; (xv) *x*+1, *y*, *z*; (xvi) −*x*+*y*+1, −*x*+1, *z*+1/3.

### Hydrogen-bond geometry (Å, °)

**Table:**

*D*—H···*A*	*D*—H	H···*A*	*D*···*A*	*D*—H···*A*
O5—H1···O4^iii^	0.83 (7)	2.09 (7)	2.878 (5)	159.80
O5—H2···O2^i^	0.81 (4)	2.09 (5)	2.845 (5)	156.

Symmetry codes: (iii) −*x*+*y*, *y*, −*z*+1/2; (i) *y*, −*x*+*y*+1, *z*+1/6.
